# Racial/ethnic differences in circulating natriuretic peptide levels: The Diabetes Prevention Program

**DOI:** 10.1371/journal.pone.0229280

**Published:** 2020-02-21

**Authors:** Deepak K. Gupta, Geoffrey A. Walford, Yong Ma, Petr Jarolim, Thomas J. Wang, for the DPP Research Group

**Affiliations:** 1 Vanderbilt Translational and Clinical Cardiovascular Research Center, Vanderbilt University School of Medicine, Nashville, TN, United States of America; 2 Division of Cardiovascular Medicine, Vanderbilt University School of Medicine, Nashville, TN, United States of America; 3 Diabetes Research Center (Diabetes Unit), Massachusetts General Hospital, Boston, MA, United States of America; 4 Department of Medicine, Harvard Medical School, Boston, MA, United States of America; 5 Center for Human Genetic Research, Massachusetts General Hospital, Boston, MA, United States of America; 6 Diabetes Prevention Program Data Coordinating Center, The Biostatistics Center, George Washington University, Rockville, MD, United States of America; 7 Biomarker Research Laboratory/TIMI Clinical Trial Laboratory, Department of Pathology, Brigham and Women’s Hospital, Boston, MA, United States of America; 8 Department of Pathology, Harvard Medical School, Boston, MA, United States of America; Ospedale del Cuore G Pasquinucci Fondazione Toscana Gabriele Monasterio di Massa, ITALY

## Abstract

Natriuretic peptides are cardiac-derived hormones that enhance insulin sensitivity and reduce fat accumulation. Low natriuretic peptide levels are associated with increased risk of type 2 diabetes mellitus (DM2); a condition with variable prevalence across racial/ethnic groups. Few studies have examined whether circulating natriuretic peptide levels and their response to preventive interventions for DM2 differ by race/ethnicity. The Diabetes Prevention Program (DPP) is a clinical trial (July 31, 1996- July 31, 2001) that randomized participants to preventive interventions for DM2. Using stored serum samples, we examined N-terminus pro-B-type natriuretic peptide (NT-proBNP) levels in 3,220 individuals (56% white; 19% African-American; 15% Hispanic; 5% American-Indian; 5% Asian). The influence of race/ethnicity on NT-proBNP concentrations at baseline and after two years of treatment with placebo, lifestyle, or metformin was examined with multivariable-adjusted regression. At baseline, NT-proBNP differed significantly by race (*P* < .001), with the lowest values in African-American individuals. Hispanic individuals also had lower baseline NT-proBNP levels compared with whites (*P*< .001), while NT-proBNP levels were similar between white, American-Indian, and Asian individuals. At two years of follow-up, NT-proBNP levels decreased in African-Americans in each of the DPP study arms, whereas they were stable or increased in the other racial/ethnic groups. In the DPP, African-American individuals had lower circulating NT-proBNP levels compared with individuals in other racial/ethnic groups at baseline and after two years of preventive interventions. Further studies should examine the cardio-metabolic implications of lower natriuretic peptide levels in African-Americans.

Trial Registration: ClinicalTrials.gov NCT00004992

## Introduction

Natriuretic peptides are cardiac-derived hormones with beneficial cardio-metabolic effects including natriuresis, diuresis, vasodilation, insulin sensitivity, and lipolysis.[1] Experimental models, genetic and epidemiologic evidence suggest that natriuretic peptide deficiencies lead to salt retention, obesity, insulin resistance, and diabetes mellitus.[2–9] Conversely, augmentation of endogenous natriuretic peptide activity seems to have beneficial effects in volume overload states such as heart failure,[10–12] while natriuretic peptide infusion lowers plasma glucose.[13]

The incidence and prevalence of cardio-metabolic diseases, such as hypertension, diabetes, and obesity vary across racial/ethnic groups; therefore, it is important to understand whether endogenous natriuretic peptide levels also differ by race/ethnicity. Though a few studies suggest that African-Americans have lower natriuretic peptide levels compared with white individuals, data from multi-ethnic cohorts are limited.[14–20] Furthermore, whether baseline differences in natriuretic peptides by race/ethnicity persist over time or change in response to intervention aimed at improving cardio-metabolic health is not known.

The Diabetes Prevention Program (DPP) is a completed clinical trial that randomized a multi-ethnic cohort of individuals at high risk for developing type 2 diabetes to receive placebo, lifestyle modification, or metformin.[21] Using stored serum samples from the DPP, we tested whether circulating N-terminus-pro B-type natriuretic peptide (NT-proBNP) differed between race/ethnic groups at baseline and after two years of intervention.

## Materials and methods

### Study population

The DPP Research Group conducted a randomized clinical trial in 27 centers in the United States between 1996–2001.[21] Inclusion and exclusion criteria, as well as the primary results, have been previously reported.[21] Briefly, eligible subjects were 25 years or older, with a body mass index ≥ 24 kg/m^2^ (≥ 22 kg/m^2^ if Asian), with evidence of impaired glucose tolerance based on elevated fasting glucose or 2 hours following an oral glucose tolerance test. Exclusion criteria were prevalent diabetes mellitus and medical conditions likely to limit life span and/or increase the risk of intervention, such as prevalent cardiovascular disease, cancer treated within the prior 5 years, creatinine ≥ 1.3 or 1.4 mg/dL in women and women, respectively or ≥ 2+ proteinuria, anemia, hepatic or other gastrointestinal diseases, recent or significant abdominal surgery, pulmonary disease with use of oxygen or bronchodilators, chronic infection, poor exercise capacity, pregnancy (recent, current, or planned), breastfeeding, major psychiatric illness, excess alcohol consumption, other endocrine disorders, hypertriglyceridemia, or use of medications (thiazide diuretics, beta-blockers, niacin, glucocorticoids, weight loss medications). In total, 3,819 participants were followed for an average of 3.2 years; 1,082 were randomly assigned to placebo; 1,079 to lifestyle; and 1,073 to metformin.[21] An additional 585 participants were assigned to troglitazone, but this arm was stopped after ten months because of concerns regarding liver toxicity.[22] The institutional review board at each center approved the protocol and all participants gave written informed consent. A list of participating centers and approving institutional review boards are provided in Table A in S1 File.

Serum samples for measurement of NT-proBNP at baseline (randomization) were available in 3,220 DPP participants. Of these 3,220 participants, 2,411 (75%) had serum samples available for measurement of NT-proBNP levels at year 2 of follow up. The discrepancy in availability of samples was related to prior sample use for other biomarker studies in the DPP as well as discontinuation of the troglitazone arm.[23]

### NT-proBNP and covariates

NT-proBNP was measured as previously described.[23] Briefly, venous blood samples were drawn from subjects according to a standardized manual of operations for the Diabetes Prevention Program. Collected blood was immediately processed at each study site with preparation of serum aliquots that were frozen initially at -20⁰C, then shipped on dry ice to the Central Biochemistry Laboratory at the University of Washington for long-term storage at -80⁰C. In 2012, serum aliquots were shipped overnight frozen on dry ice to the Biomarker Research and Clinical Trials Laboratory at Brigham and Women’s Hospital, Boston, MA. Serum samples stored at -80⁰C were thawed then centrifuged at 1,500 RPM for 5 minutes and NT-proBNP concentrations were measured using a Cobas e601 immunoanalyzer with the proBNP II assay (Roche Diagnostics, Indianapolis, IN). To reduce measurement variability, serum samples were assayed over several consecutive days. Samples with NT-proBNP concentrations below the limit of detection (< 5 ng/L; 187 at baseline, 146 at year 2) were assigned a random number between 0 and 5 ng/L. Given the skewed distribution of NT-proBNP values, natural log transformation was performed prior to entry into regression models.

Race/ethnicity was self-reported in the DPP as white, African-American, Hispanic, American-Indian, or Asian. Body mass index (BMI), blood pressure, prevalent hypertension, estimated glomerular filtration rate (eGFR) as calculated by the CKD-Epi equation, glucose and insulin were measured as reported previously in the DPP.[24, 25] For these analyses, an insulin sensitivity index was calculated as the reciprocal of HOMA-IR = (22.5/[fasting glucose (mmol/L) x fasting insulin (pmol/L)].

### Statistical analysis

Summary statistics for baseline clinical characteristics and NT-proBNP were calculated for each race/ethnic group and are presented as medians (25^th^-75^th^ percentile) or counts (percentages), for continuous and categorical data, respectively. To assess the effect of race/ethnicity on baseline natural log transformed NT-proBNP (log_e_ NT-proBNP) levels, we performed multivariable linear regression modelling with the following covariates measured at randomization: age, sex, BMI, systolic blood pressure, diastolic blood pressure, history of hypertension, eGFR, insulin sensitivity index, education, and income. From the multivariable adjusted regression models, we calculated least square means (95% confidence intervals) for log_e_ NT-proBNP and then compared these values across race/ethnic groups by analysis of variance. In order to account for multiple comparisons between race/ethnic groups, a Bonferroni corrected two-sided *P* value of < .005 was considered statistically significant. The multiplicative effects (percent difference) in NT-proBNP levels between racial/ethnic groups were calculated as (e^β^-1)*100, where β is the coefficient from multivariable adjusted linear regression models.

To assess the effect of race/ethnicity on the change in log_e_ NT-proBNP at two years, we again performed multivariable adjusted linear regression modelling. The covariates in the baseline model were also included in the two year change analysis, as were treatment group (placebo, metformin, lifestyle), baseline log_e_ NT-proBNP, and the changes in eGFR, BMI, and insulin sensitivity. The troglitazone group was not included in the change analysis given the shorter period of follow-up and smaller number of participants. An interaction term for treatment assignment by race was also included in the multivariable-adjusted models, with two-sided *P* < .05 considered significant for effect modification. The percent change in NT-proBNP levels from baseline to follow up for each race/ethnic group was calculated by exponentiating the difference in LS mean log_e_ NT-proBNP. As the two year changes in NT-proBNP levels according to randomization and race/ethnicity analysis were hypothesis-generating, two-sided *P* values < .05 were considered significant. All analyses were performed using SAS version 9.3 (SAS Institute Inc., Cary, NC).

## Results

### Baseline NT-proBNP levels

The study population was 56% white, 19% African-American, 15% Hispanic, 5% American-Indian, and 5% Asian-American. Baseline characteristics according to race/ethnicity are shown in **Table 1**. The majority of subjects were women, except among Asians. Hypertension was more common among African-Americans and Asians than the other groups.

**Table 1:** Baseline characteristics of DPP participants according to race/ethnicity.

Characteristic	White N = 1,811	African American N = 614	Hispanic N = 483	Asian N = 146	American Indian N = 166
**Male**	624 (34.5%)	157 (25.6%)	170 (35.2%)	84 (57.5%)	21 (12.7%)
**Age, years**	52 (46–60)	51 (46–58)	50 (43–57)	50 (43–58)	44 (37–51)
**BMI, kg/m**^**2**^	32.7 (28.8–37.2)	33.6 (29.7–38.8)	31.7 (28.7–35.4)	28.3 (25.7–32.2)	32.1 (29.9–36.5)
**SBP, mmHg**	123 (114–133)	127 (117–137)	121 (111–130)	126 (115–135)	113 (108–124)
**DBP, mmHg**	78 (71–84)	80 (74–87)	77 (71–84)	81 (77–90)	75 (70–80)
**Hypertension**	554 (30.6%)	231 (37.6%)	108 (22.4%)	56 (38.4%)	23 (13.9%)
**eGFR, ml/min/1.73m**^**2**^	95 (84–105)	104 (92–118)	103 (93–111)	98 (88–108)	108 (98–116)
**1/HOMA-IR**	0.17 (0.12–0.25)	0.16 (0.11–0.23)	0.15 (0.11–0.22)	0.18 (0.12–0.28)	0.16 (0.11–0.22)
**NT-proBNP, ng/L**	34.8 (17.8–64.7)	24.8 (10.2–51.1)	28.0 (13.1–50.4)	26.9 (10.8–49.8)	24.8 (14.2–40.9)
**Log**_**e**_ **NT-proBNP**	3.55 (2.88–4.17)	3.21 (2.32–3.93)	3.33 (2.57–3.92)	3.29 (2.38–3.91)	3.21 (2.65–3.71)
**Randomization**					
**Placebo**	417 (23.0%)	152 (24.8%)	106 (22.0%)	33 (22.6%)	54 (32.5%)
**Lifestyle**	496 (27.4%)	165 (26.9%)	143 (29.6%)	54 (37.0%)	58 (34.9%)
**Metformin**	551 (30.4%)	190 (30.9%)	133 (27.5%)	34 (23.3%)	51 (30.7%)
**Troglitazone**	347 (19.2%)	107 (17.4%)	101 (20.9%)	25 (17.1%)	3 (1.8%)

Values presented as median (25^th^-75^th^ percentile) and counts (percentages) for continuous and categorical data, respectively. BMI = body mass index, SBP = systolic blood pressure, DBP = diastolic blood pressure, eGFR = estimated glomerular filtration rate; HOMA-IR = homeostatic model assessment of insulin resistance; NT-proBNP = N-terminus pro B-type natriuretic peptide.

**Fig 1:**
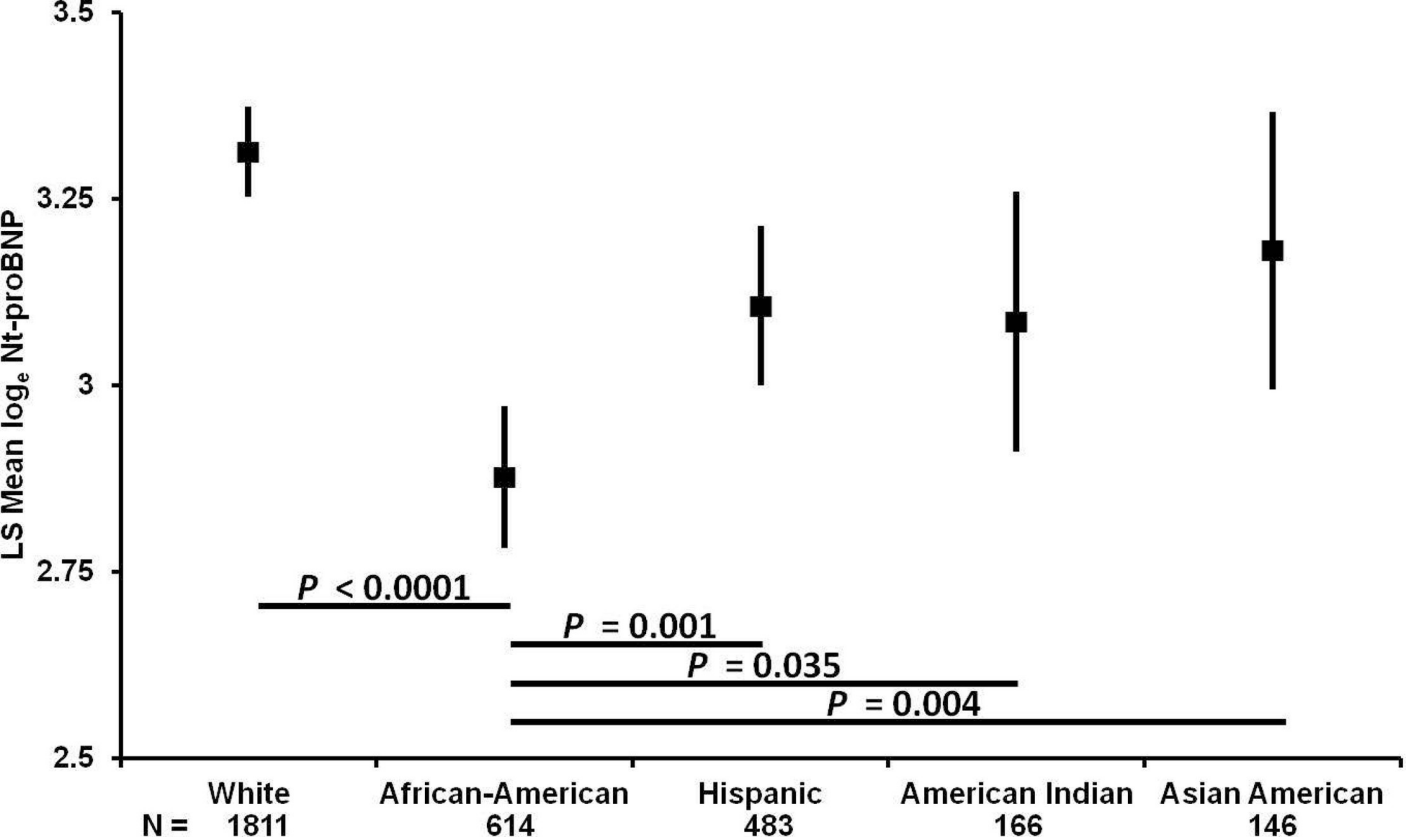
Baseline multivariable-adjusted least-square mean NT-proBNP levels in the DPP according to race/ethnicity. Least square mean log_e_ NT-proBNP values (95% confidence interval) calculated from multivariable linear regression adjusted for: age, gender, body mass index, systolic blood pressure, diastolic blood pressure, history of hypertension, insulin sensitivity, estimated glomerular filtration rate, education and income. ANCOVA *P* < .005 considered statistically significant to account for multiple comparisons.

**Fig 2:**
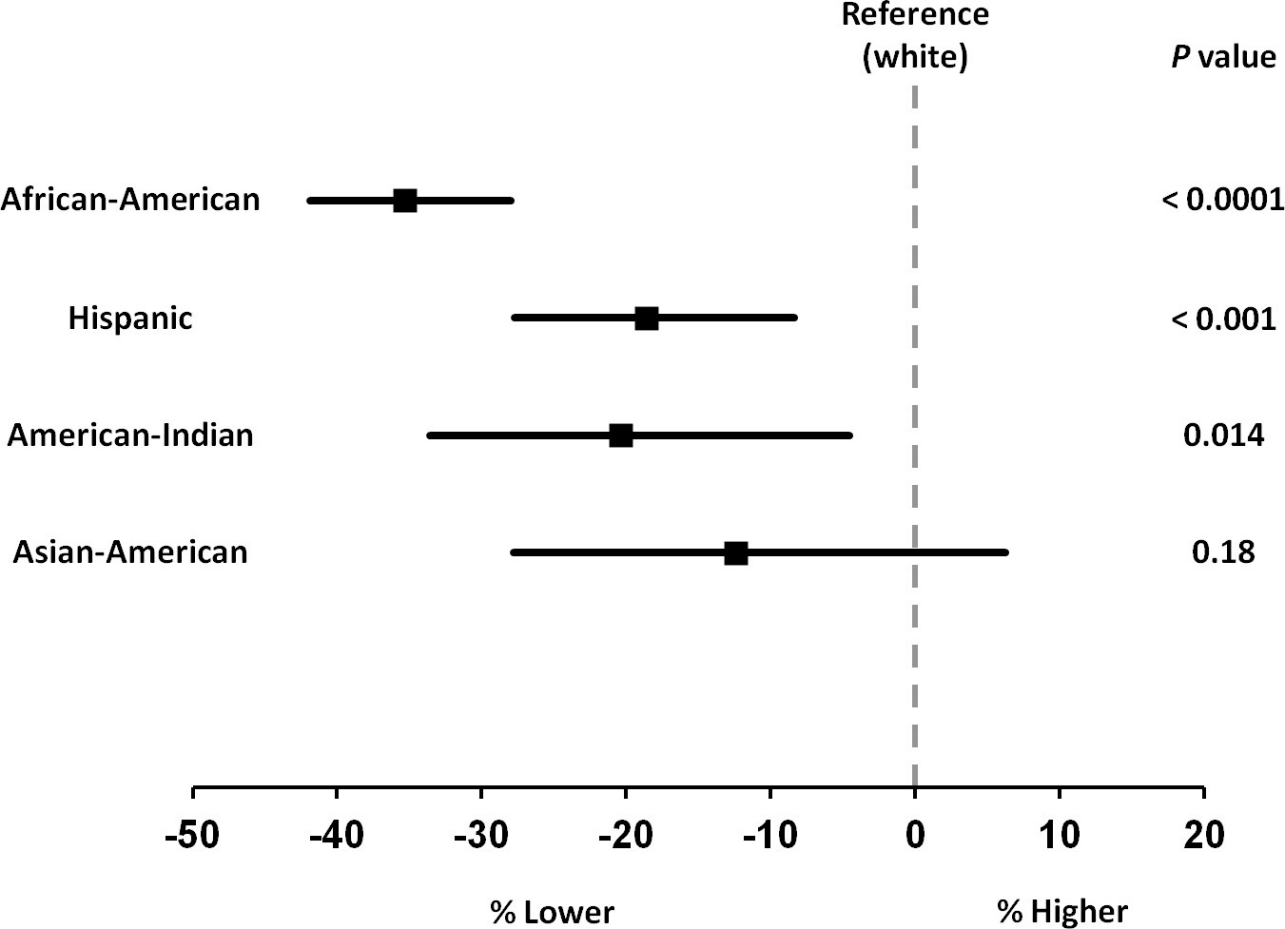
Multivariable adjusted percent differences in baseline NT-proBNP levels by race/ethnicity compared with white individuals in the DPP. Percent difference (95% confidence interval) in NT-proBNP by race/ethnicity compared with white individuals. Values calculated from multivariable linear regression adjusted for: age, gender, body mass index, systolic blood pressure, diastolic blood pressure, history of hypertension, insulin sensitivity, estimated glomerular filtration rate, education and income. ANCOVA *P* < .005 considered statistically significant to account for multiple comparisons.

Baseline NT-proBNP levels differed significantly according to race/ethnicity (*P* < .001) (**Fig 1)**. African-American individuals had lower levels than white, Hispanic, and Asian-American individuals, after adjustment for clinical characteristics (*P* < .001, .001, and .004, respectively). Hispanic individuals had lower NT-proBNP levels compared with white individuals (*P* < .001) (**Fig 2**). NT-proBNP levels were similar among white, American-Indian, and Asian-American participants.

### Two year change in NT-proBNP

**Fig 3:**
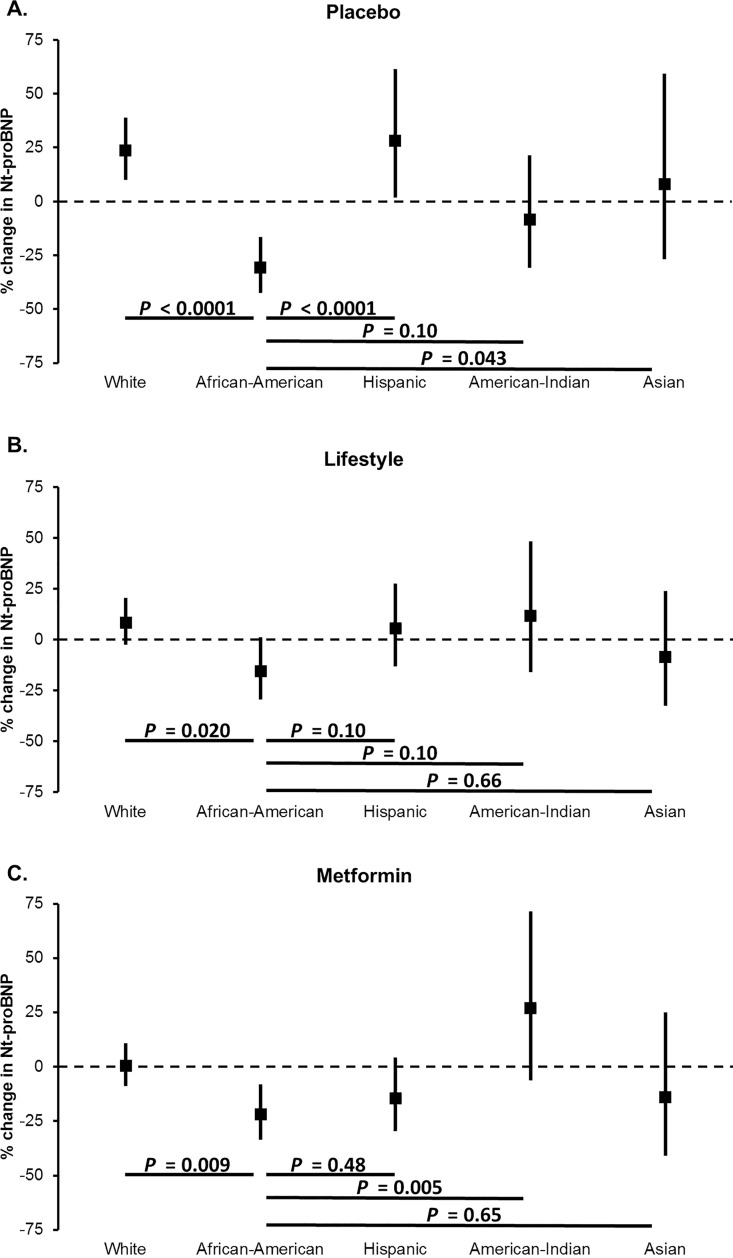
Multivariable adjusted percent change in NT-proBNP levels from baseline to two year follow up according to treatment randomization and race/ethnicity in the DPP. Percent change in NT-proBNP values (95% confidence interval) calculated from multivariable linear regression adjusted for: age, gender, body mass index, systolic blood pressure, diastolic blood pressure, history of hypertension, insulin sensitivity, estimated glomerular filtration rate, education and income, and NT-proBNP measured at baseline, as well as change in body mass index, change in insulin sensitivity, change in estimated glomerular filtration rate at 2 years of follow-up. ANCOVA *P*<0.05 considered statistically significant.

The change in NT-proBNP from baseline to 2 years was examined in multivariable linear regression with adjustment for baseline characteristics and changes in BMI, insulin sensitivity, and eGFR. Because there was a trend toward an interaction with treatment assignment (*P* = 0.06), we performed additional subgroup analyses according to treatment arm. Among participants randomized to placebo, NT-proBNP levels decreased in African-Americans, while remaining stable or increased in white, Hispanic, Asian, and American-Indian individuals (*P* < .05 for all comparisons of African-Americans vs. white, Hispanic, and Asian individuals; **Fig 3A**). Similar patterns were observed in the lifestyle and metformin groups (**Fig 3B and 3C**). The reduction in NT-proBNP in African-Americans relative to white individuals was statistically significant in both the lifestyle and metformin groups.

## Discussion

We examined differences in NT-proBNP concentrations between race/ethnic groups in the DPP at baseline and after two years of preventive interventions for type 2 diabetes. We found that natriuretic peptide levels differed significantly according to race/ethnicity, with African-Americans having the lowest levels compared with other racial/ethnic groups. This difference was independent of factors known to influence natriuretic peptide levels, including age, sex, blood pressure, BMI, and renal function. Notably, we also observed that natriuretic peptide levels in African-Americans decreased further over time relative to the other racial/ethnic groups. These trends were not explained by changes in other characteristics, such as BMI and insulin sensitivity, that are known to be influenced by the interventions in the DPP and were observed in all treatment arms, including placebo.

Few data exist from multi-racial/ethnic cohorts on the differences in circulating natriuretic peptide levels, though some studies have raised the possibility.[15–17, 20] For instance, the Biomarkers in Acute Heart Failure (BACH) trial reported lower mid-regional pro-atrial natriuretic peptide levels in Blacks compared with white individuals.[16] On the other hand, the ProBNP Investigation of acute Dyspnea in the Emergency department (PRIDE) study found that NT-proBNP levels did not differ between Black and white patients presenting to the emergency room.[14] Analyses of community dwelling participants without heart failure in the Atherosclerosis Risk in Communities, Dallas Heart Study, and Multi-Ethnic Study of Atherosclerosis support that Black individuals have lower natriuretic peptide levels compared with Whites.[8, 17–20] Our findings in the DPP, add to this existing literature by demonstrating that Black individuals have lower natriuretic peptide levels, not only compared with Whites, but also Hispanic, American-Indian, and Asian individuals. We also found that Hispanic and American-Indian individuals have lower natriuretic peptide levels compared with Whites.

We also found that racial differences in NT-proBNP levels persisted at 2 year follow-up. In particular, African-American subjects had lower NT-proBNP levels compared with white subjects not only in the placebo arm, but also in the lifestyle and metformin treatment groups. Moreover, we found in the adjusted analyses that NT-proBNP levels appeared to decline in African-American subjects from baseline to 2 year follow-up. The reasons for the relative decline in NT-proBNP levels found in African-Americans are uncertain. In our multivariable-adjusted analyses, we accounted for changes in several factors that associate with NT-proBNP levels, including body mass index, renal function, and insulin sensitivity. Residual confounding may also account for the decline in NT-proBNP levels found in African-Americans. For example, we were unable to adjust for features of cardiac structure and function, such as left ventricular ejection and mass. These features, however, would not be expected to improve over time, particularly in the placebo arm, and thus, are seemingly unlikely to account for the decline in NT-proBNP levels in African-Americans at 2 years. We were also unable to account for changes in non-trial related medications, for example anti-hypertensive medications, that could have reduced cardiovascular stress and lowered NT-proBNP levels. Nevertheless, our collective findings from baseline and 2 year follow-up lend credence to the concept that some African-American individuals may have a relative natriuretic peptide deficiency, which may have pathophysiologic consequences and therapeutic implications.

In the context of the known physiologic and metabolic actions of the natriuretic peptides, our findings in the DPP raise the possibility that reduced natriuretic peptide production could contribute to racial and ethnic differences in susceptibility to cardiometabolic disorders. A growing body of experimental evidence supports favorable metabolic actions of the natriuretic peptides.[1] For instance, murine models demonstrate that these hormones promote lipolysis, glucose sensitivity, weight loss, and energy utilization.[26] Intravenous infusions of natriuretic peptides in humans reduce glucose concentrations and induce lipid mobilization from adipose tissue, with a concomitant increase in lipid oxidation in skeletal muscle.[4, 13, 27] It follows that a primary or intrinsic natriuretic peptide deficiency may lead to increased vulnerability to cardiometabolic traits, such as hypertension, diabetes mellitus, and obesity. Data from human studies support these hypotheses. For example, in the Atherosclerosis Risk in Communities study and Multi-Ethnic Study of Atherosclerosis, low natriuretic peptide levels are associated with an increased risk of incident diabetes.[8, 9] Furthermore, individuals with genetic variants associated with lower natriuretic peptide levels appear more likely to develop hypertension, diabetes, and the metabolic syndrome.[5, 28–30] For example, genetic variants in the natriuretic peptide precursor A and B genes that result in 20% lower circulating NP levels are associated with 15% greater risk of hypertension.[28] The magnitude of this effect also suggests that the 35% lower NP levels observed in African-American compared with white individuals in the DPP have physiologic and metabolic implications.

From a clinical standpoint, identifying populations that may have reduced natriuretic peptide production may help to identify individuals most likely to benefit from novel agents that augment circulating natriuretic peptide levels, such as sacubitril-valsartan. The PARADIGM-HF trial demonstrated the superiority of combined angiotensin receptor neprilysin inhibition with sacubitril-valsartan compared with angiotensin inhibition alone in reducing heart failure hospitalizations and cardiovascular death in patients with heart failure and reduced ejection fraction, thereby supporting chronic enhancement of the natriuretic peptide system as a therapeutic strategy.[12, 31] Augmenting the natriuretic peptide system could be an attractive approach to reduce cardiometabolic risk, especially in individuals who have relative natriuretic peptide deficiencies, such as African-Americans, although this hypothesis remains to be formally tested.

The potential basis for racial/ethnic differences in natriuretic peptides is not known. Previous studies have demonstrated that natriuretic peptide levels are a heritable trait, with approximately 40% of the inter-individual variation in levels attributable to additive genetic factors.[32] It has been reported that African-Americans are more likely to carry non-synonymous variants in the *CORIN* gene compared with white individuals.[33] Corin is a cardiac serine protease that cleaves natriuretic peptide prohormones into biologically active and inactive components. Individuals with CORIN variants are at increased risk for hypertension and ventricular hypertrophy.[33] Furthermore, Black individuals are less likely to carry variants in the *NPPA* and *NPPB* genes that have been associated with increased natriuretic production.[34] For instance, the rs5068 A/G variant in the 3’ untranslated region of the *NPPA* gene is associated with higher circulating atrial natriuretic peptide levels, lower blood pressure, less hypertension, and a favorable metabolic profile.[5, 28, 35] This effect appears to be modulated by a specific micro-RNA, miR-425.[36] On the other hand, the above variants only explain a small proportion of the variability in natriuretic peptide levels.[28, 32] Thus, establishing a genetic basis for racial/ethnic differences in natriuretic peptide production would require the elucidation of a larger number of causal variants, including uncommon or rare variants.

Strengths of this study include the large sample size and multi-ethnic population that was well-phenotyped, the standardized assessment of NT-proBNP levels at both baseline and follow-up, and the adjustment for potential confounders. Limitations should also be noted. Race and ethnicity were self-reported, which may result in misclassification bias. The proportions of American-Indian and Asian subjects were relatively small, which may have limited statistical power to detect differences in NT-proBNP levels in comparisons involving those groups. In a small number of individuals, NT-proBNP levels were below the assay detection limit; however, censoring of these low levels would be expected to bias against the finding of lower natriuretic peptide levels in African-Americans. Measures of cardiac structure and function were not performed in the DPP and therefore we cannot account for differences in cardiac mass and ejection fraction that may influence racial/ethnic differences in natriuretic peptide levels. However, cardiac hypertrophy, which is associated with higher natriuretic peptide levels, is more common among African-Americans, and therefore, should bias the result towards the null.

Mature BNP was not measured in DPP because of its shorter half-life and more variable assay characteristics as compared with NT-proBNP.[37] However, NT-proBNP levels have been previously demonstrated to correlate highly with BNP levels.[38] Possible degradation of NT-proBNP due to transport and storage conditions was not specifically tested. Consequently, results for NT-proBNP levels may be underestimated due to degradation, although the relative differences in levels between race/ethnicity would be expected to be maintained as sample handling was standardized in the Diabetes Prevention Program. Furthermore, circulating NT-proBNP likely reflects natriuretic peptide production as it is synthesized in a 1:1 fashion with BNP from cardiomyocytes, but is not cleared by the same mechanisms as BNP.[39] In addition, the NT-proBNP assay used in the DPP detects both the 76-amino acid NT-proBNP as well as the full 108-amino acid prohormone (proBNP) (proBNP II, Roche Diagnostics, Indianapolis, IN), which also suggests that the observed racial/ethnic differences in circulating NP levels are largely based on differences in production.

Finally, while we examined circulating NT-proBNP levels, prior studies have demonstrated a high correlation with circulating atrial natriuretic peptide levels.[40] Recent evidence also supports that African-American individuals have lower mid-regional pro-atrial natriuretic peptide levels compared with white individuals.[16, 40] Given that the physiologic actions of atrial and b-type natriuretic peptide occur through the same guanylyl cyclase-A receptor, the beneficial metabolic effects of natriuretic peptides may to be observed with both forms.[39] Indeed, epidemiologic studies support that both lower mid-regional pro-ANP and NT-proBNP levels associate with increased risk for incident diabetes mellitus.[7, 8] Moreover, physiologic studies with infusions of ANP and BNP in humans support favorable metabolic actions of both, including lipolysis and improved glycemia.[4, 13]

## Conclusion

Among the multi-ethnic cohort of the DPP, we found that NT-proBNP levels differ significantly according to race/ethnicity, with African-American individuals having the lowest levels. These differences were maintained after two years of follow up in the DPP. Further studies should examine the cardiometabolic implications of relative natriuretic peptide deficiencies, particularly in African-Americans.

## Supporting information

S1 File (PDF)
