# Supplementary material for: Racial/ethnic differences in circulating natriuretic peptide levels: The Diabetes Prevention Program
Source: PLoS One. 2020 Feb 21;15(2):e0229280. doi: 10.1371/journal.pone.0229280 (PMC7034896; doi:10.1371/journal.pone.0229280)
Supplement: S1 File — (PDF) [file pone.0229280.s001.pdf]

SUPPLEMENTAL INFORMATION

Racial/ethnic differences in circulating natriuretic peptide levels: the Diabetes Prevention Program

Deepak K. Gupta MD MSCI, Geoffrey A. Walford MD, Yong Ma MS, Petr Jarolim MD PhD, Thomas J. Wang MD for the DPP Research Group

Contents:

1. S1 Table of IRBs
2. Research Group Investigators

S1 Table. Institutional IRBs

Institution	City	State	Name of IRB
**Pennington Biomedical Research Center**	Baton Rouge	LA	Pennington Biomedical Research Center IRB
**University of Chicago**	Chicago	IL	University of Chicago IRB
**Jefferson Medical College**	Philadelphia	PA	Thomas Jefferson University IRB
**University of Miami**	Miami	FL	University of Miami IRB
**University of Texas Health Science Center at San Antonio**	San Antonio	TX	UT Health IRB
**University of Colorado**	Denver	CO	Colorado Multiple Institutional Review Board
**Joslin Diabetes Center**	Boston	MA	Committee On Human Studies Joslin Diabetes Center
**University of Washington**	Seattle	WA	University of Washington IRB/Veterans Affairs IRB
**University of Tennessee**	Memphis	TN	University Of Tennessee Health Science IRB
**Northwestern University**	Chicago	IL	Northwestern University IRB
**Massachusetts General Hospital**	Boston	MA	Massachusetts General Hospital/Partners Human Research Committee
**University of California, San Diego**	San Diego	CA	UCSD Human Research Protections Program
**Columbia University (formerly St. Luke's-Roosevelt Hospital Center)**	New York	NY	St. Luke's-Roosevelt Hospital Center IRB/Human Research Protection Office CUMC IRB
**Indiana University**	Indianapolis	IN	Indiana University
**Medstar Research Institute**	Hyattsville	MD	Georgetown - Medstar IRB System
**University of California, Los Angeles**	Alhambra	CA	UCLA Medical IRB1
**Washington University School of Medicine**	St Louis	MO	Washington University Institutional Review Board
**Johns Hopkins School of Medicine**	Baltimore	MD	Johns Hopkins Medicine IRB1
**The University of New Mexico**	Albuquerque	NM	UNM HSC Human Research Review Committee
**Albert Einstein College of Medicine**	Bronx	NY	Albert Einstein College of Medicine
**University of Pittsburgh**	Pittsburgh	PA	University of Pittsburgh IRB
**University of Hawaii**	Honolulu	HI	University of Hawaii Human Studies Program
**SW Indian Center – Salt River/Phoenix**	Phoenix	AZ	National Institutes of Health Intramural Institutional Review Board
**SW Indian Center - Zuni**	Zuni	NM	National Institutes of Health Intramural Institutional Review Board
**SW Indian Center - Gila River**	Gila River	AZ	National Institutes of Health Intramural Institutional Review Board
**SW Indian Center - Shiprock**	Shiprock	NM	National Institutes of Health Intramural Institutional Review Board
**University of Southern California**	Los Angeles	CA	USC Office for the Protection of Research Subjects

DPP Research Group Investigators

Pennington Biomedical Research Center (Baton Rouge, LA)
George A. Bray, MD*
Iris W. Culbert, BSN, RN, CCRC**
Catherine M. Champagne, PhD, RD
Barbara Eberhardt, RD, LDN
Frank Greenway, MD
Fonda G. Guillory, LPN
April A. Herbert, RD
Michael L. Jeffirs, LPN
Betty M. Kennedy, MPA
Jennifer C. Lovejoy, PhD
Laura H. Morris, BS
Lee E. Melancon, BA, BS
Donna Ryan, MD
Deborah A. Sanford, LPN
Kenneth G. Smith, BS, MT
Lisa L. Smith, BS
Julia A. St.Amant, RTR
Richard T. Tulley, PhD
Paula C. Vicknair, MS, RD
Donald Williamson, PhD
Jeffery J. Zachwieja, PhD
***University of Chicago* (Chicago, IL)**
Kenneth S. Polonsky, MD*
Janet Tobian, MD, PhD*
David Ehrmann, MD*
Margaret J. Matulik, RN, BSN**
Bart Clark, MD
Kirsten Czech, MS
Catherine DeSandre, BA
Ruthanne Hilbrich, RD
Wylie McNabb, EdD
Ann R. Semenske, MS, RD
***Jefferson Medical College* (Philadelphia, PA)**
Jose F. Caro, MD*
Pamela G. Watson, RN, ScD*
Barry J. Goldstein, MD, PhD*
Kellie A. Smith, RN, MSN**
Jewel Mendoza, RN, BSN**
Renee Liberoni, MPH
Constance Pepe, MS, RD
John Spandorfer, MD
***University of Miami* (Miami, FL)**
Richard P. Donahue, PhD*
Ronald B. Goldberg, MD*
Ronald Prineas, MD, PhD*
Patricia Rowe, MPA**
Jeanette Calles, MSEd
Paul Cassanova-Romero, MD
Hermes J. Florez, MD
Anna Giannella, RD, MS
Lascelles Kirby, MS
Carmen Larreal
Valerie McLymont, RN
Jadell Mendez
Juliet Ojito, RN
Arlette Perry, PhD
Patrice Saab, PhD
***The University of Texas Health Science Center* (San Antonio, TX)**
Steven M. Haffner, MD, MPH*
Maria G. Montez, RN, MSHP, CDE**
Carlos Lorenzo, MD, PhD
Arlene Martinez, RN, BSN, CDE
***University of Colorado* (Denver, CO)**
Richard F. Hamman, MD, DrPH*
Patricia V. Nash, MS**
Lisa Testaverde, MS**
Denise R. Anderson, RN, BSN
Larry B. Ballonoff, MD
Alexis Bouffard, MA,
B. Ned Calonge, MD, MPH
Lynne Delve
Martha Farago, RN
James O. Hill, PhD
Shelley R. Hoyer, BS
Bonnie T. Jortberg, MS, RD, CDE
Dione Lenz, RN, BSN
Marsha Miller, MS, RD
David W. Price, MD
Judith G. Regensteiner, PhD
Helen Seagle, MS, RD
Carissa M. Smith, BS
Sheila C. Steinke, MS
Brent VanDorsten, PhD
***Joslin Diabetes Center* (Boston, MA)**
Edward S. Horton, MD*
Kathleen E. Lawton, RN**
Ronald A. Arky, MD
Marybeth Bryant
Jacqueline P. Burke, BSN
Enrique Caballero, MD
Karen M. Callaphan, BA
Om P. Ganda, MD
Therese Franklin
Sharon D. Jackson, MS, RD, CDE
Alan M. Jacobsen, MD
Lyn M. Kula, RD
Margaret Kocal, RN, CDE
Maureen A. Malloy, BS
Maryanne Nicosia, MS, RD
Cathryn F. Oldmixon, RN

* denotes Principal Investigator
** denotes Program Coordinator

Jocelyn Pan, BS, MPH
Marizel Quitingon
Stacy Rubtchinsky, BS
Ellen W. Seely, MD
Dana Schweizer, BSN
Donald Simonson, MD
Fannie Smith, MD
Caren G. Solomon, MD, MPH
James Warram, MD

VA Puget Sound Health Care System and University of Washington **(Seattle, WA)**

Steven E. Kahn, MB, ChB*
Brenda K. Montgomery, RN, BSN, CDE**
Wilfred Fujimoto, MD
Robert H. Knopp, MD
Edward W. Lipkin, MD
Michelle Marr, BA
Dace Trence, MD

University of Tennessee **(Memphis, TN)**

Abbas E. Kitabchi, PhD, MD, FACP*
Mary E. Murphy, RN, MS, CDE, MBA**
William B. Applegate, MD, MPH
Michael Bryer-Ash, MD
Sandra L. Frieson, RN
Raed Imseis, MD
Helen Lambeth, RN, BSN
Lynne C. Lichtermann, RN, BSN
Hooman Oktaei, MD
Lily M.K. Rutledge, RN, BSN
Amy R. Sherman, RD, LD
Clara M. Smith, RD, MHP, LDN
Judith E. Soberman, MD
Beverly Williams-Cleaves, MD

Northwestern University's Feinberg School of Medicine **(Chicago, IL)**

Boyd E. Metzger, MD*
Mariana K. Johnson, MS, RN**
Catherine Behrends
Michelle Cook, MS
Marian Fitzgibbon, PhD
Mimi M. Giles, MS, RD
Deloris Heard, MA
Cheryl K.H. Johnson, MS, RN
Diane Larsen, BS
Anne Lowe, BS
Megan Lyman, BS
David McPherson, MD
Mark E. Molitch, MD
Thomas Pitts, MD
Renee Reinhart, RN, MS
Susan Roston, RN, RD
Pamela A. Schinleber, RN, MS

Massachusetts General Hospital **(Boston, MA)**

David M. Nathan, MD*
Charles McKitrick, BSN**
Heather Turgeon, BSN**
Kathy Abbott
Ellen Anderson, MS, RD
Laurie Bissett, MS, RD
Enrico Cagliero, MD
Jose C. Florez, MD, PhD+
Linda Delahanty, MS, RD
Valerie Goldman, MS, RD
Alexandra Poulos

University of California-San Diego **(San Diego, CA)**

Jerrold M. Olefsky, MD*
Elizabeth Barrett-Connor, MD*
Mary Lou Carrion-Petersen, RN, BSN**
Steven V. Edelman, MD
Robert R. Henry, MD
Javiva Horne, RD
Simona Szerdi Janesch, BA
Diana Leos, RN, BSN
Sundar Mudaliar, MD
William Polonsky, PhD
Jean Smith, RN
Karen Vejvoda, RN, BSN, CDE, CCRC

St. Luke's-Roosevelt Hospital **(New York, NY)**

F. Xavier Pi-Sunyer, MD*
Jane E. Lee, MS**
David B. Allison, PhD
Nancy J. Aronoff, MS, RD
Jill P. Crandall, MD
Sandra T. Foo, MD
Carmen Pal, MD
Kathy Parkes, RN
Mary Beth Pena, RN
Ellen S. Rooney, BA
Gretchen E.H. Van Wye, MA
Kristine A. Viscovich, ANP

Indiana University **(Indianapolis, IN)**

David G. Marrero, PhD*
Melvin J. Prince, MD*
Susie M. Kelly, RN, CDE**
Yolanda F. Dotson, BS
Edwin S. Fineberg, MD
John C. Guare, PhD
Angela M. Hadden
James M. Ignaut, MA
Marcia L. Jackson
Marian S. Kirkman, MD

* denotes Principal Investigator
** denotes Program Coordinator

Kieren J. Mather, MD
Beverly D. Porter, MSN
Paris J. Roach, MD
Nancy D. Rowland, BS, MS
Madelyn L. Wheeler, RD

***Medstar Research Institute* (Washington, DC)**

Robert E. Ratner, MD*
Gretchen Youssef, RD, CDE**
Sue Shapiro, RN, BSN, CCRC**
Catherine Bavido-Arrage, MS, RD, LD
Geraldine Boggs, MSN, RN
Marjorie Bronsord, MS, RD, CDE
Ernestine Brown
Wayman W. Cheatham, MD
Susan Cola
Cindy Evans
Peggy Gibbs
Tracy Kellum, MS, RD, CDE
Claresa Levatan, MD
Asha K. Nair, BS
Maureen Passaro, MD
Gabriel Uwaifo, MD

***University of Southern California/UCLA Research Center* (Alhambra, CA)**

Mohammed F. Saad, MD*
Maria Budget**
Sujata Jinagouda, MD**
Khan Akbar, MD
Claudia Conzues
Perpetua Magpuri
Kathy Ngo
Amer Rassam, MD
Debra Waters
Kathy Xapthalamous

***Washington University* (St. Louis, MO)**

Julio V. Santiago, MD* (deceased)
Samuel Dagogo-Jack, MD, MSc, FRCP, FACP*
Neil H. White, MD, CDE*
Samia Das, MS, MBA, RD, LD**
Ana Santiago, RD**
Angela Brown, MD
Edwin Fisher, PhD
Emma Hurt, RN
Tracy Jones, RN
Michelle Kerr, RD
Lucy Ryder, RN
Cormarie Wernimont, MS, RD

***Johns Hopkins School of Medicine* (Baltimore, MD)**

Christopher D. Saudek, MD*
Vanessa Bradley, BA**
Emily Sullivan, MEd, RN**
Tracy Whittington, BS**
Caroline Abbas
Frederick L. Brancati, MD, MHS
Jeanne M. Clark, MD
Jeanne B. Charleston, RN, MSN
Janice Freel
Katherine Horak, RD
Dawn Jiggetts
Deloris Johnson
Hope Joseph
Kimberly Loman
Henry Mosley
Richard R. Rubin, PhD
Alafia Samuels, MD
Kerry J. Stewart, EdD
Paula Williamson

***University of New Mexico* (Albuquerque, NM)**

David S. Schade, MD*
Karwyn S. Adams, RN, MSN**
Carolyn Johannes, RN, CDE**
Leslie F. Atler, PhD
Patrick J. Boyle, MD
Mark R. Burge, MD
Janene L. Canady, RN, CDE
Lisa Chai, RN
Ysela Gonzales, RN, MSN
Doris A. Hernandez-McGinnis
Patricia Katz, LPN
Carolyn King
Amer Rassam, MD
Sofya Rubinchik, MD
Willette Senter, RD
Debra Waters, PhD

***Albert Einstein College of Medicine* (Bronx, NY)**

Harry Shamoon, MD*
Janet O. Brown, RN, MPH, MSN**
Elsie Adorno, BS
Liane Cox, MS, RD
Jill Crandall, MD
Helena Duffy, MS, C-ANP
Samuel Engel, MD
Allison Friedler, BS
Crystal J. Howard-Century, MA
Stacey Kloiber, RN
Nadege Longchamp, LPN
Helen Martinez, RN, MSN, FNP-C
Dorothy Pompi, BA
Jonathan Scheindlin, MD

* denotes Principal Investigator
** denotes Program Coordinator

Elissa Violino, RD, MS
Elizabeth Walker, RN, DNSc, CDE
Judith Wylie-Rosett, EdD, RD
Elise Zimmerman, RD, MS
Joel Zonszein, MD

***University of Pittsburgh* (Pittsburgh, PA)**

Trevor Orchard, MD*
Rena R. Wing, PhD*
Gaye Koenning, MS, RD**
M. Kaye Kramer, BSN, MPH**
Susan Barr, BS
Miriam Boraz
Lisa Clifford, BS
Rebecca Culyba, BS
Marlene Frazier
Ryan Gilligan, BS
Susan Harrier, MLT
Louann Harris, RN
Susan Jeffries, RN, MSN
Andrea Kriska, PhD
Qurashia Manjoo, MD
Monica Mullen, MHP, RD
Alicia Noel, BS
Amy Otto, PhD
Linda Semler, MS, RD
Cheryl F. Smith, PhD
Marie Smith, RN, BSN
Elizabeth Venditti, PhD
Valarie Weinzierl, BS
Katherine V. Williams, MD, MPH
Tara Wilson, BA

***University of Hawaii* (Honolulu, HI)**

Richard F. Arakaki, MD*
Renee W. Latimer, BSN, MPH**
Narleen K. Baker-Ladao, BS
Ralph Beddow, MD
Lorna Dias, AA
Jillian Inouye, RN, PhD
Marjorie K. Mau, MD
Kathy Mikami, BS, RD
Pharis Mohideen, MD
Sharon K. Odom, RD, MPH
Raynette U. Perry, AA

***Southwest American Indian Centers* (Phoenix, AZ; Shiprock, NM; Zuni, NM)**

William C. Knowler, MD, DrPH*+
Norman Cooeyate**
Mary A. Hoskin, RD, MS**
Carol A. Percy, RN, MS**
Kelly J. Acton, MD, MPH
Vickie L. Andre, RN, FNP
Rosalyn Barber
Shandiin Begay, MPH
Peter H. Bennett, MB, FRCP
Mary Beth Benson, RN, BSN
Evelyn C. Bird, RD, MPH
Brenda A. Broussard, RD, MPH, MBA, CDE
Marcella Chavez, RN, AS
Tara Dacawyma
Matthew S. Doughty, MD
Roberta Duncan, RD
Cyndy Edgerton, RD
Jacqueline M. Ghahate
Justin Glass, MD
Martia Glass, MD
Dorothy Gohdes, MD
Wendy Grant, MD
Robert L. Hanson, MD, MPH
Ellie Horse
Louise E. Ingraham, MS, RD, LN
Merry Jackson
Priscilla Jay
Roylen S. Kaskalla
David Kessler, MD
Kathleen M. Kobus, RNC-ANP
Jonathan Krakoff, MD
Catherine Manus, LPN
Sara Michaels, MD
Tina Morgan
Yolanda Nashboo (deceased)
Julie A. Nelson, RD
Steven Poirier, MD
Evette Polczynski, MD
Mike Reidy, MD
Jeanine Roumain, MD, MPH
Debra Rowse, MD
Sandra Sangster
Janet Sewenemewa
Darryl Tonemah, PhD
Charlton Wilson, MD
Michelle Yazzie

***George Washington University Biostatistics Center* (DPP Coordinating Center Rockville, MD)**

Raymond Bain, PhD*
Sarah Fowler, PhD*
Tina Brenneman**
Solome Abebe
Julie Bamdad, MS
Jackie Callaghan
Sharon L. Edelstein, ScM
Mary Foulkes, PhD
Yuping Gao
Kristina L. Grimes

* denotes Principal Investigator
** denotes Program Coordinator

Nisha Grover
Lori Haffner, MS
Steve Jones
Tara L. Jones
Richard Katz, MD
John M. Lachin, ScD
Pamela Mucik
Robert Orlosky
James Rochon, PhD
Alla Sapozhnikova
Hanna Sherif, MS
Charlotte Stimpson
Marinella Temprosa, MS
Fredricka Walker-Murray

***Central Biochemistry Laboratory* (Seattle, WA)**
Santica Marcovina, PhD, ScD*
Greg Strylewicz, PhD**
F. Alan Aldrich

***NIH/NIDDK* (Bethesda, MD)**
R. Eastman, MD
Judith Fradkin, MD
Sanford Garfield, PhD

***Centers for Disease Control & Prevention* (Atlanta, GA)**
Edward Gregg, PhD
Ping Zhang, PhD

+Genetics Working Group
Jose C. Florez, MD, PhD[1, 2]
David Altshuler, MD, PhD[1, 2]
Liana K. Billings, MD[1]
Ling Chen, MS[1]
Maegan Harden, BS[2]
Robert L. Hanson, MD, MPH[3]
William C. Knowler, MD, DrPH[3]
Toni I. Pollin, PhD[4]
Alan R. Shuldiner, MD[4]
Kathleen Jablonski, PhD[5]
Paul W. Franks, PhD, MPhil, MS[6, 7, 8]
Marie-France Hivert, MD[9]

1=Massachusetts General Hospital
2=Broad Institute
3=NIDDK
4=University of Maryland
5=Coordinating Center
6=Lund University, Sweden
7=Umeå University, Sweden
8=Harvard School of Public Health
9=Université de Sherbrooke

* denotes Principal Investigator
** denotes Program Coordinator
